# A Nitrile Hydratase in the Eukaryote *Monosiga brevicollis*

**DOI:** 10.1371/journal.pone.0003976

**Published:** 2008-12-19

**Authors:** Konrad U. Foerstner, Tobias Doerks, Jean Muller, Jeroen Raes, Peer Bork

**Affiliations:** European Molecular Biology Laboratory, Heidelberg, Germany; University of Pennsylvania School of Medicine, United States of America

## Abstract

Bacterial nitrile hydratase (NHases) are important industrial catalysts and waste water remediation tools. In a global computational screening of conventional and metagenomic sequence data for NHases, we detected the two usually separated NHase subunits fused in one protein of the choanoflagellate *Monosiga brevicollis*, a recently sequenced unicellular model organism from the closest sister group of Metazoa. This is the first time that an NHase is found in eukaryotes and the first time it is observed as a fusion protein. The presence of an intron, subunit fusion and expressed sequence tags covering parts of the gene exclude contamination and suggest a functional gene. Phylogenetic analyses and genomic context imply a probable ancient horizontal gene transfer (HGT) from proteobacteria. The newly discovered NHase might open biotechnological routes due to its unconventional structure, its new type of host and its apparent integration into eukaryotic protein networks.

## Introduction

Nitril hydratases (NHases, E.C. 4.2.1.84) catalyze the hydrolysis of nitriles to their corresponding amids [1]. Often, this reaction is part of a two-step degradation pathway and is followed by an amidase catalyzed step. The respective amidase converts the amid into the corresponding carboxylic acids and ammonia. The structure [2], [3] and reaction mechanism [4] of representative NHases have been extensively studied: The hetero-dimer or hetero-tetramer [2], [3] consists of two kinds of subunits - α and β - and occurs as metalloenzyme that contains either iron (non-heme Fe(III) ) or cobalt (non-corrin Co(III)) ions [5]–[8]. The biological function of the NHases is unknown so far but it was shown that they enable the respective organism to utilize aliphatic, aromatic and hetero-aromatic nitriles as sole nitrogen source under laboratory conditions e.g. [9], [10]. Due to their ability to selectively and efficiently hydrolyze cyano groups, NHases are heavily used in biotechnological industry e.g. for the synthesis of the essential chemicals acrylamide (30,000 tons/year [11]) and nicotinamide (>3500 tons/year [12]). In addition, their enzymatic activities are used to remove toxic nitriles (e.g. nitrile herbicides) during waste water treatment [13].

So far, NHases are described to occur in species belonging to the phyla Proteobacteria, Actionobacteria, Cyanobacteria and Firmicutes, in habitats ranging form soil [14], via costal marine sediments [15] and deep sea sediments [10], [16] to geothermal environments [17], [18]. Here, using a large scale screen for NHases in public sequence databases and metagenomic datasets, we describe the identification of the first eukaryotic NHase and investigate its origin.

## Results

In order to get an overview about the phylogenetic and habitat distribution of NHases, we created HMMs (Hidden-Markov-Model) for each of the two subunits based on 42 α and 48 β subunit sequences and screened 12,126,382 proteins (or protein fragments) from UniRef and seven metagenomic data sets from diverse environments. In total, 324 α (including 14 of thiocyanate hydratases (SCNases) [19]) and 265 β (including 4 SCNases) subunit members were found in this homology search step. The α subunit HMM seems to be more sensitive when applied to fragmented sequences – the ratio of α to β sequences is not 1∶1 as expected (for fully sequenced genomes, this ratio is obtained; see Table S1). Yet, the HMMs identify both subunits in most of the species in UniRef that harbor NHases and also in some of the metagenomic scaffolds.

**Figure 1:**
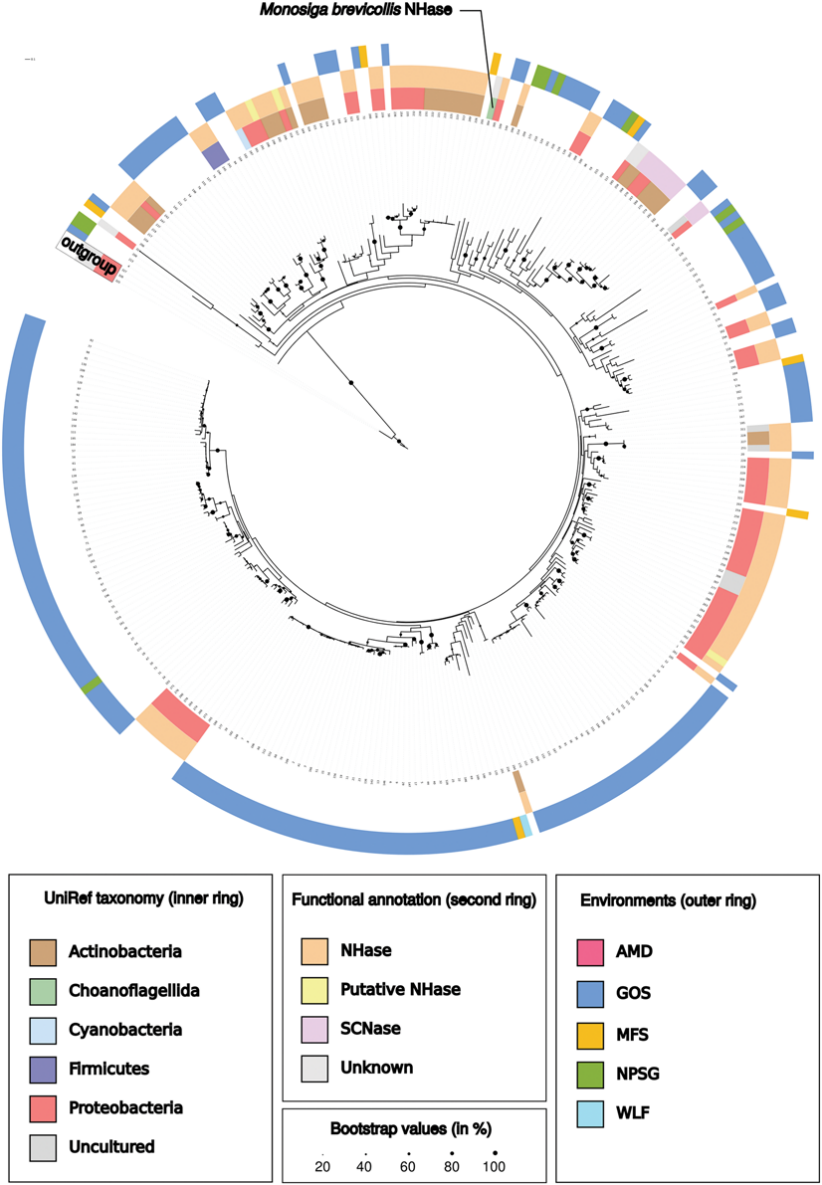
Maximum-likelihood tree of the NHase α subunit sequences. (AMD – acid mine drainage, MFS – Minnesota farm soil, GOS - Global Ocean Sampling Expedition, NPSG - North Pacific Subtropical Gyre, WLF – whale falls). The *Monosiga* sequence clusters together with sequences from GOS, MFS, NPSG and Actinobacteria and Proteobacteria from UniRef. A large fraction of GOS sequences form a separated branch (weak bootstrap support) with different subgroups. All these sequences seem to originated from Proteobacteria as our BLAST-based analysis indicate (Methods S1). The β subunit shows a similar trend .

To confirm the NHases membership of the identified sequences, to study the taxonomic distribution of the originating organisms and to possibly define new subgroups we constructed maximum likelihood trees of both subunits. These trees (Figure 1) confirmed that the detected sequences are NHases and show taxonomic clustering. They illustrate that all sequences – also the metagenomic ones - seem to originate from bacterial species, with a large fraction of proteobacterial NHases found in the Global Ocean Sampling Expedition dataset (Table S1 and Figure S1). There is one notable and surprising exception to this observation: both subunits are contained in a single hypothetical open reading frame (UniProt identifier A9V2C1) of the recently sequenced choanoflagellate *Monosiga brevicollis* [20], as deposited in the UniRef database.

The unicellular *Monosiga brevicollis* is one of more than 125 known choanoflagellates which represent the closest known relatives of metazoans (i.e. are closer to animals than plants and fungi). They can form simple multicellular colonies and are found in marine, brackish and freshwater habitats in which they use their apical flagellum to prey bacteria [21].

As *Monosiga* would be the first eukaryote that harbors an NHase, we analyzed the respective gene and encoding protein in detail.

**Figure 2:**
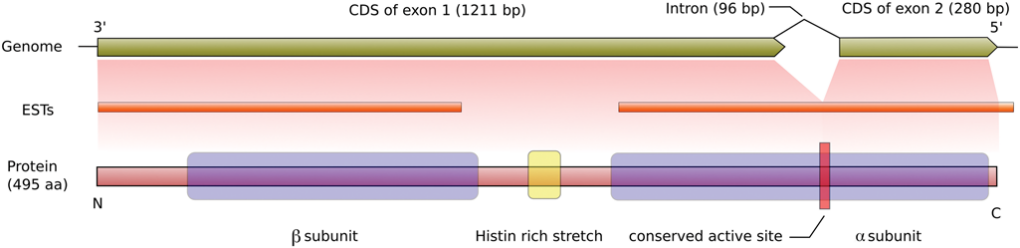
Scheme of the genomic region, ESTs and the protein of the NHases in *Monosiga brevicollis.* The β subunit and the Histidin-rich stretch are located in the protein part coded by the CDS of exon 1 while the α subunit consist of coding parts of exon 1 and exon 2. The putative active site is pinpointed in the α subunit and its coding sequence contains an intron in that site. The two ESTs confirm the expression of both subunits and prove the splicing of the intron.

The putative NHase is 496 amino acids long and contains the usually separately encoded subunits fused into one protein connected by a Histidin-rich stretch (Figure 2). Both subunits seem complete and the putative ion binding active site in the α subunit (single letter code: CXXCSC) that is necessary for NHase functioning [1] appears conserved. The orientation of the two subunits in the coding region of the genome of Monosiga brevicollis is different from the operon structure in most bacteria; the β subunit is located 5′-terminal, the α subunit 3′-terminal while in bacteria the domains are usually arranged in the order α- β (5′ to 3′). The phylogenetic analysis (Figure 1) shows that the protein clusters together with NHases of proteobacterial origin and a BLAST-based analysis clearly indicates proteobacteria as the most similar homologs (Methods S1 and Methods S2).

In order to exclude contamination and check for likely functionality, we analyzed genomic features and EST (expressed sequence tag) data. The expression of the gene is strongly supported by the existence of two ESTs covering a large portion of the gene (Figure 2). Furthermore, one EST (accession number JGI_XYM3899.rev) implies that the gene contains a 96 bp long intron in the active site. The GC value of the corresponding transcripts (59.4%) differs only slightly from the median GC value of all *Monosiga* transcripts (56.9%) which strengthen the assumption that it is a gene of *Monosiga* and not bacterial contamination of the genome sequence.

Putative amidases could be detected with HMMs in *Monosiga*'s protein set (as in other eukaryotes) but their genes are distantly located to the NHase in the genome and show only low similarity to the NHase-connected amidases in bacteria. Despite the fact that the identified amidases do not seem to be transferred from a proteobacterial donor together with the NHase, it is possible that an existing *Monosiga* amidase took over this functionality but we cannot exclude that the NHase products are processed differently in this choanoflagellate.

## Discussion

The discovery of an NHase in an eukaryote, i.e. *Monosiga brevicollis*, from a sister group of animals, indicates a wider phylogenetic spread of NHases than currently believed. The presence of an intact domain structure, an (EST supported) intron and the similarity between the GC content of the gene and the surrounding genomic sequence makes a bacterial contamination extremely unlikely. As the eukaryotic NHase has a phylogenetic position within diverse bacterial NHases (Figure 1), the currently most parsimony explanation is that it resulted from an ancient horizontal gene transfer from bacteria into the choanoflagellate or a more ancient eukaryotic lineage. As it has been sustained for a considerable time to allow for GC amelioration, NHase functionality must have provided a selective advantage. The HGT hypothesis is corroborated by the absence of the sequence in any sequenced lower eukaryote so far, as well as the presence of highly repetitive stretches less than 10 bp upstream (5′) of the gene which could have served as a site for homologous recombination and insertion of this gene. This hypothesis would need an additional inversion event to have occurred after the HGT to change the subunit order (see Results). As the alternative explanation (its presence at the root of all eukaryotes combined with multiple, independent losses in various eukaryotic lineages) is less parsimonious, we tend to think HGT is the most likely explanation of the observed results.

Unfortunately, we are unable to predict the natural substrate of *Monosiga's* NHase and the low concentrations of nitriles expected in its habitats will likely hamper the determination of the precise role of the NHase in the physiology and ecology of this organism. For some aquatic bacteria, nitriles were previously reported to serve as nutritional sources [15], [16], [22]. We observe NHases in all samples of the Global Ocean Sampling Expedition and most samples of the North Pacific Subtropical Gyre implying a general ecological and nutritional importance of this enzyme. Here we hypothesize that Monosiga has acquired the functionality to utilize nitriles for nutritional purposes.

From the biotechnological perspective, this newly discovered nitrile hydratase might be of relevance, too. The enzyme with fused subunits and a different type of host might have beneficial features like higher activity, higher stability or new substrate specificities.

## Materials and Methods

### Data sets used

In this study sequences from the UniRef100 database [23] and the full set of proteins of *Monosiga brevicollis* [20] (downloaded from the JGI web site www.jgi.doe.gov) were analyzed. Additionally, we screened predicted proteins from the following metagenomics samples: Minnesota farm soil [24], Global Ocean Sampling Expedition [25], human gut flora [26], acid mine drainage [27], enhanced biological phosphorus removal sludges [28], North Pacific Subtropical Gyre [29] and whale falls (sunken whale bones) [24].

### HMM creation

To create highly selective and specific Hidden-Markov-Models (HMM) of the two NHase subunits, available HMMs were retrieved from Pfam [30] (accession PF02979.7 and PF02211.6) and used for searches with *hmmsearch* (part of the HMMER package [31]) against the UniRef100 protein set. The extracted sequences were aligned with the program *muscle* [32]. Based on these manually cleaned alignments (Methods S2), we constructed and calibrated HMMs (Methods S3).

### HMM search, tree construction and visualization

The UniRef and metagenomics protein data sets were screened by *hmmsearch* with the two NHase HMMs. After that the detected sequences were aligned with *hmmalign* (also included in the HMMER package). We manually added outgroup sequences to the alignments. The programs phyml [33], *clann* [34] and *seqboot* (PHYLIP packages [35]) constructed two trees (with 100 bootstrap repetitions) (Methods S4) based on these alignments. After that Python scripts (www.python.org) (Methods S5 - available as open source under the ISC license (http://www.opensource.org/licenses/isc-license.txt)) integrated the sequence and taxomic information, annotation strings, trees and HMM search data into a database (Methods S6 - availability under the Creative Commons Attribution License (http://creativecommons.org/licenses/by/3.0/)) and created coloring files for iTOL [36] to visualize the trees (Methods S4).

### Species mapping of environmental sequences

To map sequences from *Monosiga brevicollis* and metagenomic data sets to species a BLAST-based placing method was applied (Methods S1 and Methods S2).

### Manual analysis

The manual analysis of the genomic region was performed with the tools *Artemis* [37] and *Clustal X* [38].

## Supporting Information

Table S1 Number of sequences detected with NHase specific HMMs.(Abbreviations: AMD = Acid mine drainage; EBPRS = Enhanced biological phosphorus removal sludges; GOS = Global Ocean Sampling expedition; HGUT = Human gut flora; MFS = Minnesota farm soil; NPSG = North Pacific Subtropical Gyre; WLF = Whale falls (sunken whale bones)); There were no significant HMM hits in AMD, EBPRS and HGUT.
(0.02 MB PDF)

Methods S1 Monosiga NHase species mapping in visualized iTOL.
(0.05 MB PDF)

Methods S2 Protein alignments of the the Monosiga NHase and other NHase domains
(0.01 MB ZIP)

Methods S3 HMM files
(0.03 MB ZIP)

Methods S4 Tree files and coloring files for the NHase α and β domain search results.
(0.38 MB ZIP)

Methods S5 Python scripts for the data analysis
(0.02 MB ZIP)

Methods S6 Database files - availability under the Creative Commons Attribution License (http://creativecommons.org/licenses/by/3.0/)
(0.11 MB ZIP)

Figure S1 A. Number of sequences detected with NHase specific HMMs in the different data set. B. Ratio of detected á and â sequences in the different data set.
(2.51 MB TIF)
