# Supplementary material for: A Nitrile Hydratase in the Eukaryote Monosiga brevicollis
Source: PLoS One. 2008 Dec 19;3(12):e3976. doi: 10.1371/journal.pone.0003976 (PMC2603476; doi:10.1371/journal.pone.0003976)
Supplement: Table S1 — Number of sequences detected with NHase specific HMMs.(Abbreviations: AMD = Acid mine drainage; EBPRS = Enhanced biological phosphorus removal sludges; GOS = Global Ocean Sampling expedition; HGUT = Human gut flora; MFS = Minnesota farm soil; NPSG = North Pacific Subtropical Gyre; WLF = Whale falls (sunken whale bones)); There were no significant HMM hits in AMD, EBPRS and HGUT. (0.02 MB PDF) [file pone.0003976.s001.pdf]

Sample	α	β
UniRef	108	83
GOS	199	170
NPSG	9	5
MSF	7	4
WLF	1	3
