# Supplementary figures and images for: A Nitrile Hydratase in the Eukaryote Monosiga brevicollis
Source: PLoS One. 2008 Dec 19;3(12):e3976. doi: 10.1371/journal.pone.0003976 (PMC2603476; doi:10.1371/journal.pone.0003976)

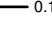

Supplement: Methods S1 — Monosiga NHase species mapping in visualized iTOL. (0.05 MB PDF) [file pone.0003976.s002.pdf]

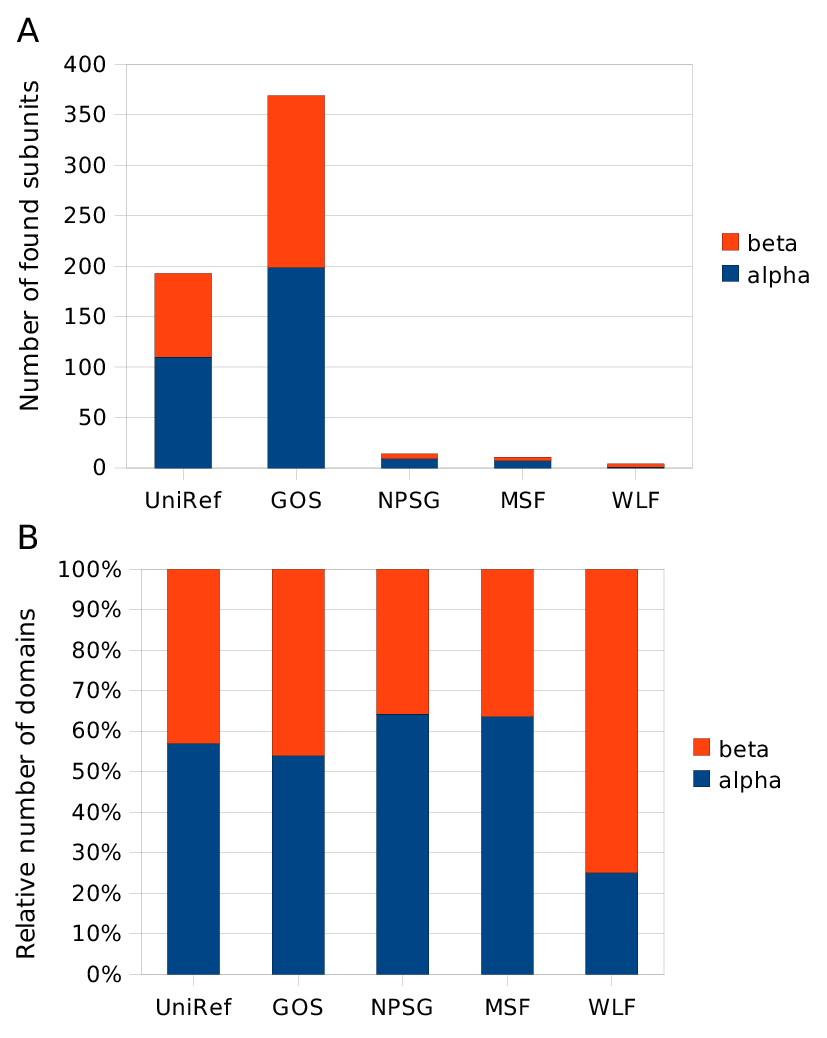

Supplement: Figure S1 — A. Number of sequences detected with NHase specific HMMs in the different data set. B. Ratio of detected á and â sequences in the different data set. (2.51 MB TIF) [file pone.0003976.s008.tif]
