# Supplementary material for: A Nitrile Hydratase in the Eukaryote Monosiga brevicollis
Source: PLoS One. 2008 Dec 19;3(12):e3976. doi: 10.1371/journal.pone.0003976 (PMC2603476; doi:10.1371/journal.pone.0003976)
Supplement: Methods S4 — Tree files and coloring files for the NHase α and β domain search results. (0.38 MB ZIP) [file pone.0003976.s005.zip › Tree_files/NHase_alpha_tree.pdf]

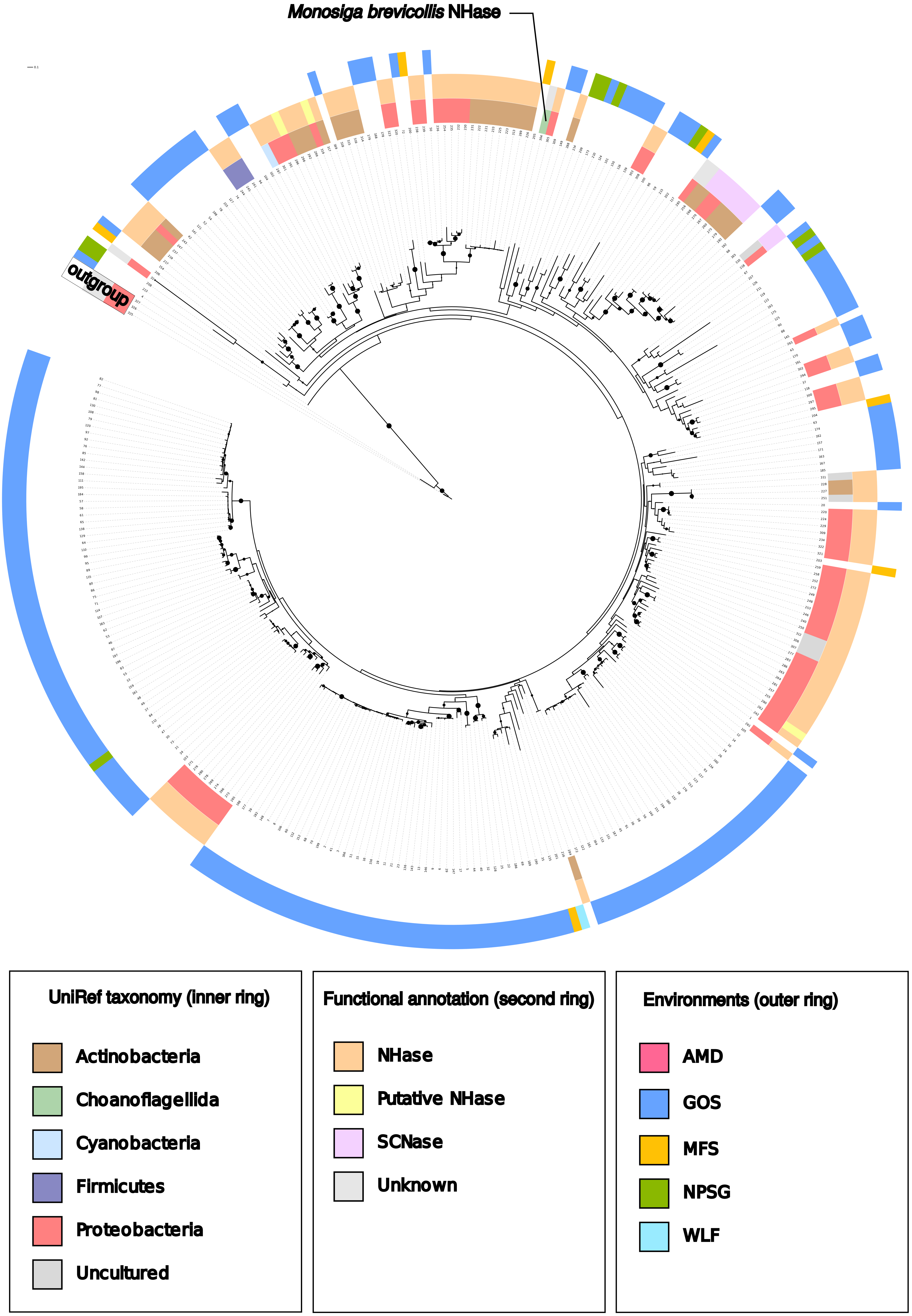

Monosiga brevicollis NHase
outgroup
UniRef taxonomy (inner ring)
Actinobacteria
Choanoflagellida
Cyanobacteria
Firmicutes
Proteobacteria
Uncultured
Functional annotation (second ring)
NHase
Putative NHase
SCNase
Unknown
Environments (outer ring)
AMD
GOS
MFS
NPSG
WLF
